# HybridQC: A SNP-Based Quality Control Application for Rapid Hybridity Verification in Diploid Plants

**DOI:** 10.3390/genes15101252

**Published:** 2024-09-26

**Authors:** Patrick Obia Ongom, Yakub Adebare Ajibade, Saba Baba Mohammed, Ibnou Dieng, Christian Fatokun, Ousmane Boukar

**Affiliations:** 1 International Institute of Tropical Agriculture (IITA), Kano 713103, Nigeria; ajibadeyakubadebare@gmail.com (Y.A.A.); s.mohammed@cgiar.org (S.B.M.); o.boukar@cgiar.org (O.B.); 2 International Institute of Tropical Agriculture (IITA), Ibadan 200001, Nigeria; i.dieng@cgiar.org (I.D.); c.fatokun@cgiar.com (C.F.)

**Keywords:** software program, quality assurance, quality control, hybridity determination, F_1_ verification, parental purity, KASP assay, single nucleotide polymorphism, marker efficiency

## Abstract

**Background/Objectives**: Hybridity authentication is an important component of quality assurance and control (QA/QC) in breeding programs. Here, we introduce HybridQC v1.0, a QA/QC software program specially designed for parental purity and hybridity determination. HybridQC rapidly detects molecular marker polymorphism between parents of a cross and utilizes only the informative markers for hybridity authentication. **Methods:** HybridQC is written in Python and designed with a graphical user interface (GUI) compatible with Windows operating systems. We demonstrated the QA/QC analysis workflow and functionality of HybridQC using Kompetitive allele-specific PCR (KASP) SNP genotype data for cowpea (*Vigna unguiculata*). Its performance was validated in other crop data, including sorghum (*Sorghum bicolor*) and maize (*Zea mays*). **Results:** The application efficiently analyzed low-density SNP data from multiple cowpea bi-parental crosses embedded in a single Microsoft Excel file. HybridQC is optimized for the auto-generation of key summary statistics and visualization patterns for marker polymorphism, parental heterozygosity, non-parental alleles, missing data, and F_1_ hybridity. An added graphical interface correctly depicted marker efficiency and the proportions of true F_1_ versus self-fertilized progenies in the data sets used. The output of HybridQC was consistent with the results of manual hybridity discernment in sorghum and maize data sets. **Conclusions:** This application uses QA/QC SNP markers to rapidly verify true F_1_ progeny. It eliminates the extensive time often required to manually curate and process QA/QC data. This tool will enhance the optimization efforts in breeding programs, contributing to increased genetic gain.

## 1. Introduction

Molecular markers have become indispensable to breeding, given the need to accelerate the rate of genetic gain to meet the growing global food demand. For several past centuries, the human population depended entirely on the outcome of conventional breeding efforts for food and other aesthetic needs. The human population is projected to reach 9.8 billion by 2050 [1], and the resultant food demand has provoked a fundamental shift in how crop breeding is being conducted. There is unanimous agreement on fully integrating genomics into the breeding process to achieve the desired speed and genetic gain [2,3,4]. This comes with the need to restructure breeding programs to accommodate molecular marker applications, including the capacity to handle large volumes of molecular data often used in making breeding decisions. Molecular marker technologies have advanced over time from hybridization-based restriction fragment length polymorphisms (RFLPs) to PCR-based random-amplified polymorphic DNA (RAPD), amplified fragment length polymorphisms (AFLPs), and sequence repeats (SSRs), and more recently, high-throughput single-nucleotide polymorphisms (SNPs) [5]. Recent advances that have lowered the cost of high-throughput sequencing technology have led to the development of several genotyping platforms [6,7]. These developments have significantly changed the approach to marker discovery and analyses. To cut the cost even further, low-density genotyping platforms have been developed with few and cheap markers for easy and routine breeding applications. One such platform is the KASP assay technology, which is based on the fluorescence resonance energy transfer and allele-specific oligo extension system [8,9]. Low-density KASP assays have been used in different crop species, including cowpea (*Vigna unguiculata*) for hybridity testing and parental fingerprinting [10], sorghum (*Sorghum bicolor*) for marker-assisted introgression [11], and Cassava (*Manihot esculenta*) for marker-assisted selection [12], among others. It is evident that molecular markers are now being deployed across many scientific fields, including developmental biology, systematics, conservation biology, and forensic studies [13]. In modern plant breeding, molecular markers are pivotal in constructing genetic maps, identifying underlying gene traits, studying genetic variability, and quality assurance (QA) and quality control (QC). 

Quality assurance focuses on well-established processes and standards that prevent mixing high-quality germplasm with low-quality or genetically impure materials [14]. A good QA therefore prevents mistakes in developing or maintaining new breeding lines. Quality control, on the other hand, aims to identify and correct errors or mixers that might have slipped through QA protocols [14,15]. Typically, QC allows for checking the true genetic identity of parental lines relative to the original source, determining seed genetic purity, and verifying whether the hybrids are truly derived from the specified parents, among other applications [14]. QA/QC is therefore a fundamental aspect of breeding optimization efforts aimed at minimizing errors and wastage of resources and time in breeding operations [10,14,16], thereby enabling increased genetic gains [17]. Breeding is a multi-stage, costly, and time-consuming intervention with the end goal of generating high-quality varieties acceptable to the end users. Therefore, deploying molecular markers for QA/QC at critical breeding stages would ensure precise selection decisions, and the correct type of genetic materials are carried forward in the breeding program. Diagnostic markers for QA/QC have been developed in several crops and are being used to address different QA/QC aspects, including genetic fingerprinting and purity of the parental germplasm, parent–offspring identity, genetic purity of hybrids, validation of crosses in nurseries, and tracking specific traits in germplasm [10,11,12,14,16,17,18,19].

Marker deployment for routine QA/QC in a breeding program also needs efficient means of processing the data to facilitate faster decisions. Despite recent progress in developing diagnostic markers for QA/QC in crop breeding, limited applications can process low-density marker panels. Consequently, QA/QC data are often manually scrutinized in Microsoft Excel, which becomes daunting when large data volumes are to be handled. Efforts to address this gap led to the development of one QC application called Flapjack v1.22.04.21, a graphical genotyping software that can perform marker-assisted backcrossing, forward breeding, and pedigree verification, among other functions [20]. Several different molecular software programs do exist but are not explicitly designed for QA/QC in a breeding program; these include applications for genetic diversity analysis [21,22,23], polymorphism analysis [5,24], marker-assisted recurrent selection [25], and QTL discovery analysis [26,27]. We introduce HybridQC, a QA/QC software program designed explicitly for F_1_ verification in diploid species. HybridQC computes genetic polymorphism between parental lines and uses only polymorphic markers to assess the genuineness of purported F_1_ offspring. This allows breeders to discard selves by clearly discriminating homozygotes (accidental selves) from plants that are expected to be heterozygous. We have demonstrated the functionalities of HybridQC using KASP-based SNP data from the cowpea breeding program at the International Institute of Tropical Agriculture (IITA). Users can download HybridQC installation executables for Windows and example data from GitHub at https://github.com/Ayatoo047/HybridQC-build/releases/tag/v1.0.0 (accessed on 24 September 2024).

## 2. Materials and Methods

### 2.1. The Genotype Data

We used KASP-based SNP genotype data from IITA’s cowpea breeding program to test the functionality of the software. The data consist of 1408 putative F_1_ offspring plus 206 parental lines that were genotyped with 22 QA/QC SNP markers. The F_1_s were developed by making pair-wise crosses among 206 parental lines, giving rise to 103 bi-parental populations. The number of F_1_ offspring per bi-parental population ranged from 5 to 34. Tissue samples were collected from individual F_1_ offspring and their parents using the protocol previously described by [10]. DNA extraction and genotyping were conducted at the Intertek lab in Sweden following the company’s KASP assay genotyping protocol. Genotyping was conducted based on KASP assays for the 22 cowpea markers (17 QA/QC SNP panel + 5 diagnostic markers). The profiles of the cowpea QA/QC SNP panels were previously described and are publicly available [10]. The genotyping output in Intertek format has been provided as an example of data in the Supplementary Materials.

### 2.2. Software Development

**Figure 1:**
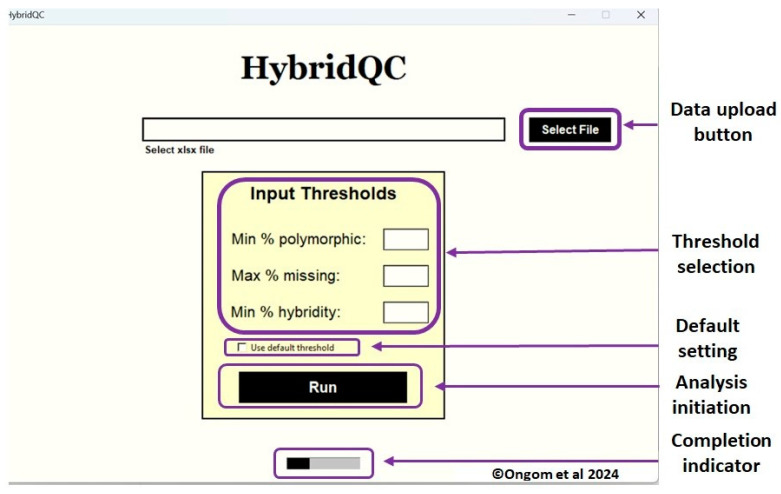
The GUI application window of HybridQC. It depicts different functionalities including data uploading, threshold settings, and the run buttons.

HybridQC was written in Python language. Python is a trendy programming environment because it can be implemented in various ways, including automation, website development, and data analysis. Python scripts were written and optimized to read direct KASP assay genotyping output in Intertek format. To make the software user-friendly without the need to understand Python programming, we created a graphical user interface (GUI) application that is easily installable on Microsoft Windows operating systems. The executable file is supplied in this article as Supplementary Materials. The GUI is equipped with widgets for easy navigation. These widgets include a file upload button, threshold selection buttons with default settings, and analysis and run completion buttons (Figure 1). The threshold selection of HybridQC allows the user to set three key thresholds: minimum acceptable level of polymorphism, maximum allowable level of missing data, and hybridity. By default, hybridity is determined only if marker polymorphism between parents is ≥20% and missing data is ≤20%; otherwise, “NA” is returned to indicate a lack of confidence in assessing the authenticity of the F_1_ offspring. In addition, an offspring is considered true F_1_ if hybridity ≥ 50%; otherwise, “FALSE” is returned to indicate possible self-fertilization. When the default setting is not selected, the user can set their threshold levels.

### 2.3. The Genetic and Mathematical Principles

**Figure 2:**
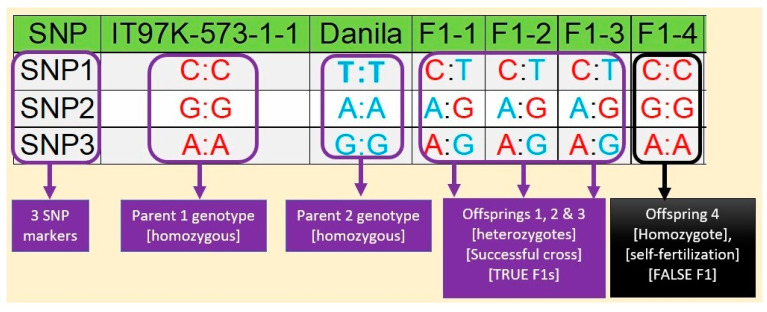
An illustration of the molecular discernment of hybridity in cowpea. Molecular markers are represented by three SNPs (SNP1, SNP2, and SNP3). The F1s were derived from two parents: an IITA variety IT97K-573-1-1 and a land race Danila. The alleles of parents IT97K-573-1-1 and Danila are presented in red and blue fonts, respectively, and the parents are homozygous across all three SNP marker loci. The purported four F_1_ offspring are shown as F1-1, F1-2, F1-3, and F1-4. Offspring numbers 1 to 3 are heterozygous at all three SNP marker loci and are considered true F1s while offspring number 4 has homozygous alleles at all 3 loci, and the alleles are similar to parent 1; hence, it is a product of self-fertilization and is considered false F_1_.

The application was developed to analyze the genuineness of the first-generation offspring coming from a cross between two parents. In diploid species like cowpea, an offspring receives one allele each from the two parents. Molecular markers can track these alleles in the offspring, making it possible to discern if an offspring is a true cross of the specified parents. An illustration of offspring hybridity detection using molecular markers in cowpea is presented in Figure 2. HybridQC was designed to imitate this detection process and summarize the hybridity results.

To effectively determine the hybridity of a purported F_1_ offspring, a marker must first differentiate between the two parents in what is termed polymorphism detection. If a molecular marker cannot distinguish between the two parents, it is referred to as monomorphic and is considered uninformative. This application detects and computes marker polymorphism based on the formula described by [10]:

(1) $$Parental\;Marker\;Polymorphism=\left(\frac{Pm}{Tm-Mc}\right)\times 100$$

where Pm is the number of polymorphic markers per pair of parents, Tm is the total number of markers used to genotype the pairs of parents, and Mc is the number of missing genotype calls in the two parents of a cross.

The application selects only highly polymorphic markers and uses these marker sets to determine the hybridity of individual F_1_ offspring. Percent hybridity is then computed based on the formula modified from [10] to account for missing genotype calls:

(2) $$Hybridity=\left(\frac{L_{het}}{Pm-Mc}\right)\times 100$$

where $L_{het}$ is the number of polymorphic SNPs detecting an F_1_ as heterozygous (true hybrid), Pm is the number of all the polymorphic SNPs between the parents of a particular F_1,_ and Mc is the number of missing genotype calls. 

HybridQC is also sensitive to highly heterozygous parental lines, and hence, it computes parental heterozygosity. Hybridity results of F_1_ offspring from highly heterozygous parents are considered invalid since the genotypes of a true hybrid from such parents are impossible to decode. The percentage of heterozygous loci for each parent is computed as shown:

(3) $$Parental\;Heterozygosity=\left(\frac{P_{het}}{T_{m-Mc}}\right)\times 100$$

where $P_{het}$ is the number of parental loci that are heterozygous, $T_{m}$ is the total number of SNP markers, and Mc is the number of missing genotypes calls in the two parents of a cross.

The software is also capable of detecting non-parental alleles in the F_1_ offspring. The offspring are scanned for the presence of strange alleles, which is indicative of either outcrossing, seed mixture, or sometimes genotyping errors. The percentage of non-parental alleles for each F_1_ offspring is computed as shown:

(4) $$Non-parental\;Alleles=\left(\frac{L_{npa}}{Pm}\right)\times 100$$

where $L_{npa}$ is the number of loci that have non-parental (strange) alleles, and $P_{m}$ is the number of polymorphic SNP markers.

We also added functionality to assess the performance of the markers based on the ability to differentiate between parental lines, referred to here as molecular marker efficiency. Marker efficiency is assessed based on the formula [10]:

(5) $$Marker\;efficiency=\left(\frac{fm}{Tc}\right)\times 100$$

where fm is the frequency of marker polymorphism among parental pairs, and Tc is the total number of parental combinations.

### 2.4. Assumptions

HybridQC analyzes data from co-dominant genetic markers, especially SNPs, with genotype data conforming to the Intertek KASP assay output. Other types of co-dominant markers would have to be re-coded to match the KASP assay genotype data formats. HybridQC analysis assumes that the species is diploid and that markers are autosomal. It also assumes that markers are inherited independently of each other, in other words, that they are in linkage equilibrium. Consequently, HybridQC operates on well-developed QA/QC marker panels selected based on good genome coverage, high SNP polymorphism, and SNP call neutrality. 

## 3. Implementation

### 3.1. The Analysis Workflow

**Figure 3:**
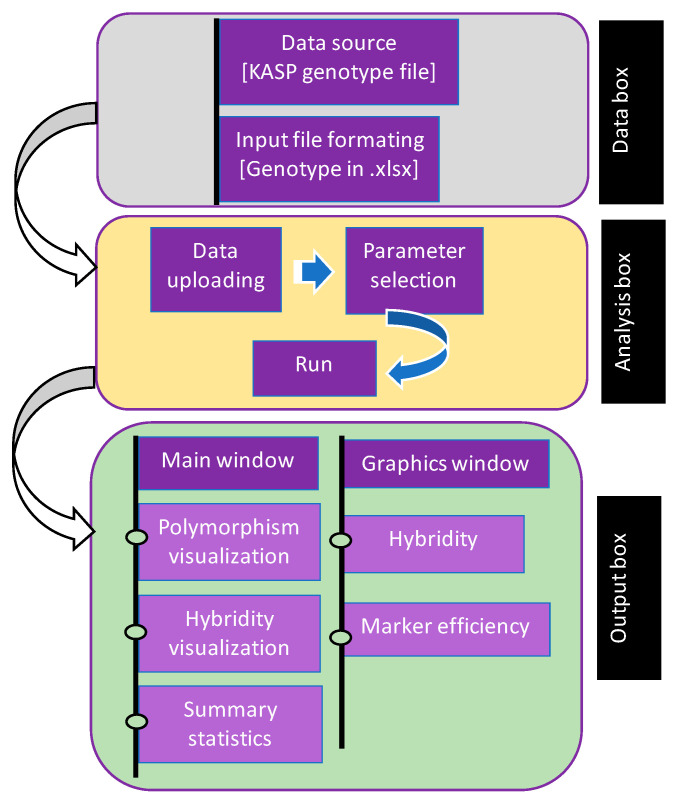
QA/QC analysis workflow from HybridQC software.

The workflow for QA/QC analysis using HybridQC has been presented in Figure 3. The workflow has three components: (i) Data box, which involves the acquisition of KASP assay SNP data and putting it in the right format; (ii) Analysis box, which is equipped with functionalities to upload the SNP data, select the threshold parameters, and run the analysis; and (iii) Output box, which is divided into the main window and graphics window. The main window contains the analysis results with polymorphisms and hybridity color hits. In addition, columns with summary statistics are added in this window. The graphic window is generated in new sheets, and it contains a pie chart and bar chart for hybridity and marker efficiency, respectively (Figure 3).

### 3.2. The Input Data and Analysis

**Figure 4:**
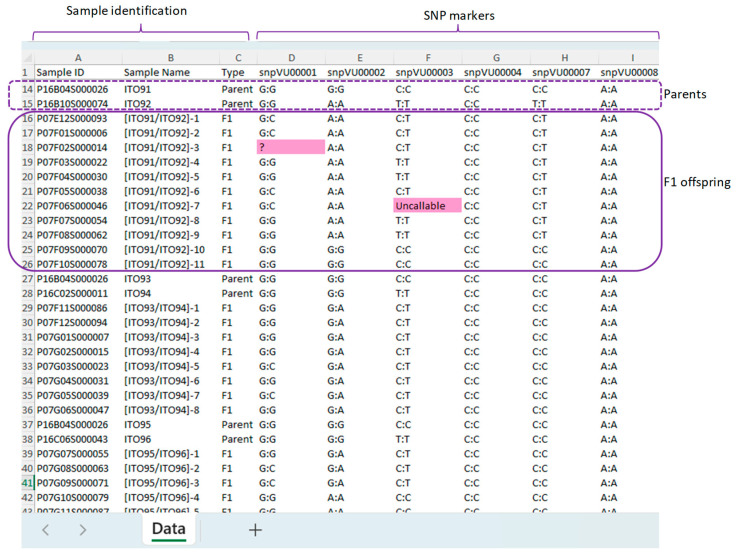
Example input data format recommended for HybridQC. The application accepts data in the “.xlsx” file extension. The first 3 columns must have the “Sample ID”, “Sample Name”, and “Type” while the remaining columns are for the SNP markers. The cells containing “?” and “uncallable” highlighted in pink depict the acceptable symbols for missing SNP calls. The rectangular dash and solid lines indicate the genotypes of parents and the offsprings respectively.

The input data are a direct result of the KASP assay in Intertek format. It contains the SNP genotype arranged into groups, starting with the two parents of a cross followed by the derived F_1_ offspring (Figure 4). Multiple bi-parental crosses can be included in one input data file. The input data file is formatted to include a column labeled “Sample ID”, a unique label that identifies the sample. The second column contains the “Sample Name”, which specifies the pedigrees or the actual names of the samples, and the third column is designated as “Type”, which identifies each sample either as a parent or an F_1_ offspring. The remaining columns contain the SNP marker IDs. Each SNP marker has two alleles represented by a combination of any of the four DNA nucleotide bases: A, T, C, and G, that are used to score the genotype of all samples. The missing SNP calls are represented by the symbol “?” or “Uncallable” (Figure 4). A typical example of input data are provided in Supplementary Materials. 

Data analysis is accomplished first by clicking the file selection button and browsing the Microsoft Excel input data, which should have been saved in a computer directory with the file extension “.xlsx”. This is followed by setting the desired input thresholds including minimum polymorphism, maximum missing data, and minimum hybridity levels; otherwise, a default threshold is selected, and the analysis is completed by clicking the “Run” button. 

### 3.3. HybridQC Output

**Figure 5:**
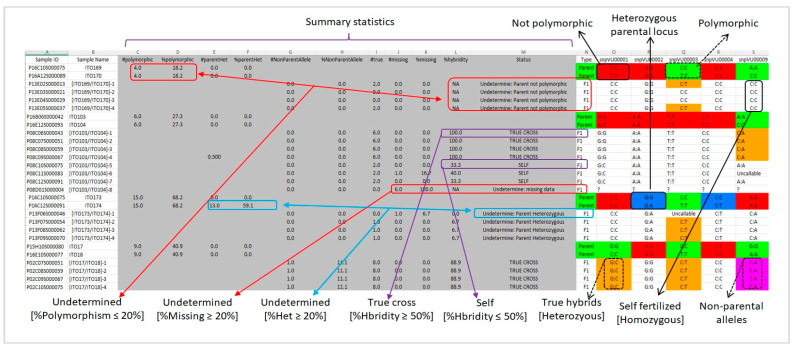
HybridQC analysis output shows the summary statistics and color pattern depiction of polymorphism, parental heterozygosity, and hybridity. Columns A and B contain Sample ID and Sampe Name, Columns C-M contain the summary statistics, Column N contains the germplasm type (Parent vs. F_1_), and Columns O-S contain the marker genotype calls. Red cells indicate non-polymorphic parental loci while green represent polymorphic loci. The blue cells depict parental loci that are heterozygous. The orange cells represent loci that detected offsprings as being heterozygous. The purple cells show detection of non-parental alleles. The boxes and the respective arrows highlight and explain the type of output generated by HybridQC. “NA” indicate that %hybridity was not computed.

Parental polymorphism: Following a successful analysis, HybridQC generates output in an Excel spreadsheet that presents the results in the form of color patterns, graphical visualization, and statistical summaries. The first output is the pattern of SNP polymorphism between the parental pairs and parental heterozygosity. The polymorphism pattern is depicted as color hits with the polymorphic SNP alleles between the parents marked in green while the non-polymorphic alleles are red (Figure 5). This identifies the informative SNP markers that would be considered to test the authenticity of purported F_1_ offspring, while the monomorphic (uninformative) SNPs are excluded from subsequent analysis. The pattern of parental heterozygosity is represented by blue cells depicting loci that are heterozygous for at least one of the two parents involved in a cross. Highly heterozygous parents are considered impure, and therefore, the computation of hybridity is ignored for the resultant F_1_ offspring. 

Hybridity status: The second output is the pattern of hybridity status of the offspring, which is also presented as color hits with orange cells delineating SNP genotypes that identify an offspring as a true hybrid (Figure 5). These loci are heterozygous for the unique alleles coming from the two parents. However, the cells containing homozygous alleles are left uncolored and they represent the product of self-fertilization (failed cross). In addition, HybridQC is able to detect F_1_ offspring that carry non-parental alleles. This is depicted by purple cells in the output (Figure 5).

Summary statistics: The third key output includes summary statistics that are inserted as new columns with the headings “#polymorphic”, “%polymorphism”, “#parentHet”, “%parentHet”, “#NonParentAllele”, “%NonParentAllele”, “#true”, “#missing”, “%missing”, “%hybridity”, and “status” (Figure 5). The columns for “#polymorphic” and “%polymorphism” present the computations for counts and percentages of polymorphic SNP markers, respectively, between each parental combination. The columns for “#parentHet”, and “%parentHet” present the statistics for counts and percentage marker loci that are heterozygous, respectively, in each parental line. The column for “#true” presents the results for counts of marker loci that are heterozygous and therefore detect an offspring as a true hybrid. The “#NonParentAllele” and “%NonParentAllele” columns contain results for counts and percentages of loci, respectively, that have non-parental (strange) alleles. On the other hand, “#missing” and “%missing” depict counts and percentages of missing SNP genotype calls, respectively. The column labeled “%hybridity” presents the computation for the level of the hybridity of an offspring expressed in percent. It takes the frequency (counts) of polymorphic SNP loci, detecting an offspring as a true hybrid, and expresses it as a percent of the total number of polymorphic loci. Another column labeled “status” generates five types of feedback comments: (i) “TRUE CROSS”, implying the offspring is a true hybrid, (ii) “SELF”, referring to self-fertilized offspring, (iii) “Undetermine: missing data”, meaning there is insufficient data to draw a conclusion regarding hybridity of offspring, (iv) “Undetermine: Parent not polymorphic” implying the number of informative SNPs is insufficient to draw a conclusion regarding hybridity of offspring, and (v) “Undetermine: Parent Heterozygous” indicating that hybridity of an F_1_ offspring is obscured by the level of heterozygosity of the parents (Figure 5). These comments depend on the thresholds that can be set during the analysis and are meant to provide easy decision making on the selection of true offspring to advance in the breeding program. By default, if the %hybridity is ≤50%, a status “SELF” is returned; otherwise, it is “TRUE CROSS”. In addition, if %polymorphism is ≤20% and %missing ≥20%, the status “Undetermined: Parent not polymorphic” and “Undetermined: missing data” are returned, respectively. The user is provided with the option to change these default settings by unselecting the default input thresholds and manually inserting their desired thresholds. The threshold for parental heterozygosity is set at a maximum of 20% by default, such that, the hybridity status of offspring derived from parents with heterozygosity exceeding 20% is labeled “Undetermine: Parent Heterozygous”.

**Figure 6:**
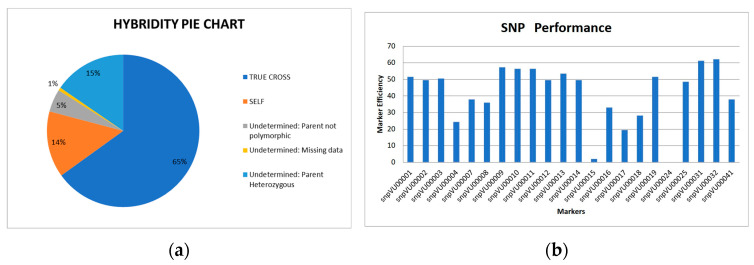
HybridQC graphical interface. (**a**) A pie chart showing the proportions of F_1_ offspring that were identified as true hybrid (TRUE CROSS), self-fertilized (SELF), and the proportion undetermined due to lack of polymorphism among parents, excessive missing data, and high parental heterozygosity. (**b**) A bar graph presenting the performance of each QC SNP marker based on the ability to differentiate between the parents. On the *x*-axis are the SNP markers, and the *y*-axis is the marker efficiency.

Visualization: In addition, HybridQC automatically generates some graphics that allow us to easily summarize the hybridization results. A pie chart of hybridity and SNP maker efficiency is populated in separate sheets during the analysis. The hybridity pie chart presents the proportion of purported F_1_ offspring that are identified as being true hybrids and those that are products of self-fertilization. In the case of the example data from cowpea, 73% of all F_1_ offspring were true hybrids, while 21% were failed crosses (Figure 6a). The pie chart also presents the proportions of offspring whose authenticity cannot be determined due to a lack of sufficient information, particularly related to marker polymorphism and missing data. In the example of the cowpea data set, the undetermined proportions due to lack of polymorphism and missing data were 5% and 1%, respectively (Figure 6a). HybridQC also assesses each SNP marker’s efficiency, allowing the opportunity to evaluate future usage of these markers. The example data showed that the SNP marker “snpVU00015” was less efficient than others in differentiating between the parents (Figure 6b).

### 3.4. Validation of HybridQC Performance

We created 12 data scenarios to evaluate the performance of HybridQC under different data sizes, with the number of SNPs ranging from 22 to 202 and F_1_ samples varying from 104 to 4831 (Supplementary File S4). HybridQC processed a data set of 104 to 1000 samples genotyped with 22 QA/QC SNP SNPs within 0.4 to 1 s. Supplying data size 100 SNPs by 2500 samples, the software computation time increased to 60 s, while with a combination of 202 SNPs and 4831 samples, the run time was 360 s (Supplementary File S4). When we compared this output with that from manually processed data, it revealed a 100% match in the hybridity and parental purity results. In addition, we validated the performance of the software using published sorghum (*S. bicolor*) quality control SNP data [28] and maize (*Zea mays*) [29]. The sorghum data consisted of 39 putative F_1_ progenies derived from two bi-parental crosses (Supplementary File S5), while the maize data had 87 F_1_ derived from five bi-parentals (Supplementary File S6). Using default settings, HybridQC could accurately reproduce published hybridity results that were manually computed (Supplementary Files S7 and S8). For instance, out of 39 sorghum F_1_ samples, the manually assessed hybridity status of 38 (97%) samples matched the results from HybridQC. One sample (KARIMTAMA1 × FRAMIDA_17) was registered by the software as “undetermined” because the sample exceeded the missing data threshold (Supplementary File S7), an outcome that was not detected manually. For the maize data set, published hybridity results were 100% equivalent to the results from HybridQC (Supplementary Files S8). 

## 4. Discussion

Quality control and assurance are fundamental in every breeding program as they are the basis for ensuring operational efficiency in terms of resource use and time. It is an aspect of breeding optimization efforts that is key in enhancing genetic gain [17]. The realization of the significance of an efficient QA/QC system in breeding programs has sparked the development of low-density diagnostic markers suitable for this purpose. In most cases, it is difficult to distinguish crop germplasm or identify genetic impurities morphologically or biochemically. Molecular markers are abundant, unaffected by the environment, and highly discriminative [30], making them fit for QA/QC in crop plants. DNA-based markers like SNPs are ideal for genetic testing because they are highly polymorphic, co-dominant in expression to allow effective differentiation between homozygotes and heterozygotes, are highly reproducible, expressed at all the developmental stages, have known positions in the genome, often also linked with traits of interest, and possible to automate so that one can handle thousands of samples in quick time [14,30]. Because of these qualities, applying molecular markers for QA/QC in breeding programs is becoming routine. In the CIMMYT maize breeding program, a detailed molecular marker-based QA/QC protocol was developed for routine deployment in parental selection, parentage verification of maize hybrids, genetic purity, identity, and reference profiles of finished inbred lines and breeder’s seeds [14]. In cowpea, a 17 QA/QC SNP panel was developed and validated for routine use in parental fingerprinting, germplasm purity profiling, and F_1_ hybridity verification [10]. Recently, the authors of [28] validated 49 quality control KASP SNPs for sorghum and demonstrated their usage in hybridity determination. Similar advances are reported in root and tuber crops such as yam (*Dioscorea* spp.) [31] and sweet potato (*Ipomoea batatas*) [17]. It should be noted that molecular marker integration into breeding programs requires the capacity to process and manage the data. This necessitates the development of appropriate tools to process molecular data to facilitate decision-making. This need has long been realized, as evidenced in the development of many software programs, including linkage mapping and QTL analysis [26,32], genome-wide association mapping [33,34,35], population genetic diversity [21,22,23], and genomic predictions [36,37,38]. These are significant advances in bridging the data processing gap in molecular integrated breeding; however, these software programs address only the trait discovery needs, leaving the post-discovery aspects wanting. After the discovery and validation of QTL, specialized markers tagging the QTL region are often designed for routine deployment in breeding. These include trait markers that are deployed either in forward breeding or marker-assisted backcrossing (MABC) and QA/QC markers. To enhance the QA/QC data analysis workflow in breeding programs, we developed HybridQC, a program that efficiently processes KASP-based SNP data for F_1_ hybridity verification in diploid species. A limited number of software programs are designed specifically for QA/QC in plant breeding. One such software is an Online Marker Efficiency Calculator (iMEC v1.0) [5] that is limited only to the computation of marker performance based on polymorphism information content (PIC). In addition, OptiMAS v1.5 [25], was also designed specifically for marker-assisted recurrent selection, allowing for tracking parental alleles and selecting the best parents for intermating. One software close enough to HybridQC, that incorporates several types of molecular data analysis, including pedigree verification of F_1_ progeny to verify the trueness of a cross, marker-assisted backcrossing, and forward breeding, is the Flapjack [20]. Unlike Flapjack, HybridQC is devoted to determining the genuineness of putative F_1_s, and it does this with high efficiency without the need for complicated file formats and analysis. Flapjack requires the creation of map and genotype files, and all bi-parental population data are handled separately. HybridQC can process the genotype data of putative F_1_ progenies of multiple bi-parental populations at once, without the need to create independent data sets for each bi-parental cross. Consequently, HybridQC analyzes thousands of F_1_ progenies from multiple crosses in a single run. Performance tests with different data sizes revealed that HybridQC can analyze more than 4000 samples genotyped with 200 QA/QC SNPs within 360 s. In most cases, QA/QC marker panels are not more than 50 SNPs which can be processed by HybridQC within 10 to 30 s depending on the sample size. Further evaluation of software performance using data sets from sorghum [28] and maize [29] depicted results consistent with published manually computed data. The maize data provided an example of ideal data with no missing calls and all putative F_1_s being true hybrids. The sorghum data were a typical example of common breeding data, with 3% missing genotype calls and 28% false hybrids. The software accurately detected these patterns, validating its effective performance in different crops. HybridQC accepts genotypes directly in Intertek format, eliminating the need for multiple and complex file format conversions often required by other software programs. However, if the user obtains QA/QA SNP data from other genotyping platforms in VCF and other formats, we recommend using other software programs such as PGDSpider [39] and TASSEL [33] for data conversion. The results are simple to interpret, accompanied by graphical summaries and visualization patterns. HybridQC generates the output in Excel spreadsheets with simple statistical summaries of the percentage hybridity of purported F_1_ and a feedback comment that allows quick selection of the desired offspring to advance in the breeding program.

## 5. Conclusions

A simple and specialized software program was developed for hybridity authentication in diploids. Currently, there are several software tools for molecular analyses, and they have different functionalities, providing computation solutions for diverse genetic and genomic studies. Exceptionally few programs are designed to process low-density molecular data that are deployed for QA/QC in plant breeding programs. Breeders have no option but to manually curate marker data, resulting in delays in making breeding decisions. HybridQC provides a user-friendly platform to conduct F_1_ verification analysis using SNP markers. Future upgrades of HybridQC are hoped to accommodate other QA/QC aspects such as genetic purity, genetic identity, and reference profiling. This application will strengthen QA/QC programs in breeding operations and facilitate the effective use of markers as part of modernization efforts for increased genetic gain.

## Data Availability

All data reported in this study has been provided as Supplementary Materials. In addition, the installation executables for HybridQC, for example, data and source codes can be accessed on GitHub at https://github.com/Ayatoo047/HybridQC-build/releases/tag/v1.0.0 (accessed on 24 September 2024).

## Supplementary Materials

The following supporting information can be downloaded at https://www.mdpi.com/article/10.3390/genes15101252/s1. File S1: software installation executables for Microsoft Windows; File S2: Example cowpea input data containing KASP-based SNP genotypes in Intertek format; File S3: Example output from the analysis of cowpea SNP data using HybridQC software; File S4: Results of performance evaluation in large data sets; File S5: sorghum data set; File S6: Maize data set; File S7: Hybrid QC results from sorghum data; File S8: HybridQC results from maize data.
